# The Effectiveness of Art Therapy on Children and Adolescents with ASD: A Systematic Review of RCTs

**DOI:** 10.3390/healthcare13222960

**Published:** 2025-11-18

**Authors:** Shijuan Wei, Angel Hor Yan Lai, Howard Wing Hong Ho

**Affiliations:** Department of Applied Social Sciences, The Hong Kong Polytechnic University, Hong Kong 999077, China

**Keywords:** autism spectrum disorder, art therapy, music therapy, drama therapy, systematic review, children and adolescents, randomized controlled trials

## Abstract

Background: Autism Spectrum Disorder (ASD) is a neurodevelopmental condition that affects social interaction, communication, and behavior. Traditional interventions such as Applied Behavior Analysis and sensory integration therapy often lack a person-centered approach. Art therapy offers a creative and holistic alternative for supporting children and adolescents with ASD. Objectives: This study systematically reviewed and evaluated the effectiveness, modalities, formats, and methodological quality of randomized controlled trials (RCTs) involving art therapy interventions for children and adolescents with ASD. Methods: A systematic search of eight databases identified 12 RCTs involving art therapy for children and adolescents with ASD. Intervention outcomes, formats, and methodological rigor were assessed through this systematic review. Methodological rigor was assessed using the Cochrane ROB 2.0 tool, and the Delphi list with four additional items. Results: Art therapy showed promise in reducing ASD symptoms and stress-related symptoms, and in improving social communication, motor skills, language, and neurodevelopment. Most studies had limitations, including small sample sizes, short durations, a high risk of bias, and low methodological quality. Conclusions: Although existing studies suggest that art therapy may benefit children and adolescents with ASD, further rigorously designed studies are required to establish its efficacy and inform evidence-based practice.

## 1. Introduction

Autism Spectrum Disorder (ASD) is a neurodevelopmental condition characterized by persistent deficits in social communication and restricted, repetitive patterns of behavior [1]. Children and adolescents with ASD may experience difficulties in understanding social-emotional reciprocity, as well as in verbal and nonverbal communication. They often have impaired abilities to develop, maintain, and comprehend relationships, alongside ritualized behavior patterns, abnormal intensity or focus of interests, and hypersensitivity or hyperreactivity to sensory input [1,2,3,4]. ASD affects approximately 0.6% to 0.72% of children and adolescents worldwide from an early age, with recent meta-analyses indicating an increasing prevalence over recent decades [1,2,3,4]. ASD can lead to functional impairments in various settings, thus qualifying it as a disability. In school, children and adolescents with ASD frequently struggle with adhering to rules, achieving academic success, and interacting with peers, placing them at a higher risk of being bullied [5,6]. In community settings, they may encounter challenges in organizing or planning their daily activities [7]. These discrepancies between the external environment and the characteristics of ASD can result in frustration and anxiety among them, negatively impacting their and their caregivers’ psychological well-being [8].

Intervention for ASDs encompasses a variety of approaches targeting different socio-behavioral symptoms. These treatments include Applied Behavior Analysis (ABA), Cognitive Behavioral Therapy (CBT), Sensory Integration Therapy (SIT), Early Start Denver Model (ESDM), and therapies focusing on social and professional skills [9]. While these interventions have demonstrated effectiveness, each comes with its own limitations. ABA, despite its widespread use and evidence-based success, has been criticized for being too rigid and overly focused on compliance rather than fostering intrinsic motivation and personal autonomy [10]. Therapies focusing on social skills and occupational training can be highly effective but may fall short if not tailored to the individual’s needs and often struggle with generalization to real-world settings [11]. Sensory integration therapy is criticized for focusing on symptoms rather than the holistic needs [12]. Overall, while these approaches show benefits, their effectiveness can be limited in generalizing to real-life situations, and a lack of emphasis on empowerment and holistic health. In response, practitioners have increasingly adopted more person-centered and developmentally appropriate approaches, such as art therapy. However, empirical evidence supporting the effectiveness of art therapy for children and adolescents with ASD remains limited [13,14,15,16]. To address this gap, the present study investigates the impact of art therapy on the psychological, social, and behavioral outcomes of children under the age of 18 diagnosed with ASD.

### 1.1. Benefits of Art Therapy on Children and Adolescents with ASD

Art therapy is a holistic therapeutic approach that integrates principles from mental health, human development, and psychological theory with various art modalities—such as visual art, music, movement, drama, and creative writing—to promote individual and community well-being [17,18]. Art therapy adopts approaches from psychodynamic therapy, humanistic approaches, and family therapy, and its key features include using art (e.g., drawing, music, drama) to facilitate psychological healing; symbolic expression; client-centered and strength-based practice; and nonverbal and multisensory experience [18]. With these features, art therapy can effectively promote emotional expression by externalizing inner experiences in a safe and symbolic form; facilitate self-discovery and strengthen self-efficacy; alleviate stress and anxiety; and encourage positive coping strategies support. It can also foster interpersonal interaction and communication, particularly in group settings [19].

Art therapy may support children and adolescents with ASD in sensory processing, attention abilities, flexibility, motor skills, social learning, and emotional expression [20]. In art therapy, therapists create safe, rich and supportive sensory experiences for children to facilitate sensory integration and processing [21]. By “looking together”, during the art-making process, art therapy enhances joint attention ability and further fosters social learning and attachment [22]. In art therapy, children and adolescents are typically encouraged to create artwork freely by themselves, which can increase behavioral flexibility, strengthen their motor skills, and promote relaxation in daily environments [20]. As children and adolescents with ASD often struggle to identify and verbally express emotions, art therapy can serve as a safe medium for emotional expression and support mental well-being [20,23].

### 1.2. A Theoretical Framework for Art Therapy in ASD

Beyond the potential benefits described above, and considering the psychoanalytic and humanistic foundations of art therapy [17,18,24], this paper proposes Self-Determination Theory (SDT) as a conceptual framework to better understand the mechanisms underlying the effectiveness of art therapy for individuals with ASD [25,26,27,28].

SDT posits that psychological well-being is supported by the fulfillment of three fundamental needs: autonomy, competence, and relatedness. **Autonomy**—the experience of acting with volition and self-endorsement—is considered the central psychological need within SDT. Research indicates that environments fostering autonomy, such as classrooms and therapeutic settings, are associated with increased engagement, reduced anxiety, improved emotional regulation, and enhanced social and academic outcomes [26,28,29]. **Competence** refers to the sense of effectiveness and mastery in one’s activities, while **relatedness** involves forming meaningful interpersonal connections. A growing body of evidence supports positive associations between competence, relatedness, and psychosocial well-being in both neurotypical children and those with ASD [28,30,31].

**Figure 1:**
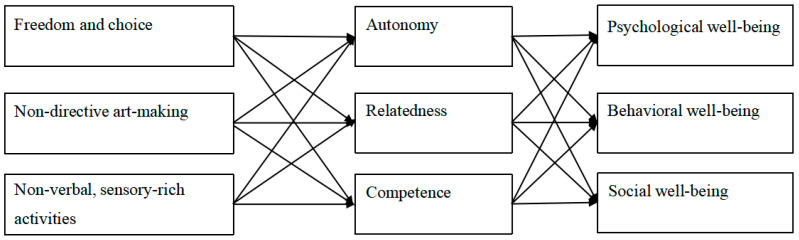
Art Therapy, Self Determination Theory and Psychosocial and Behavioral Outcomes.

Art therapy establishes conditions that support the fulfillment of psychological needs, thereby enhancing the psychological, social, and behavioral outcomes of children with ASD. Its defining characteristics include: (a) **freedom and choice**, which foster autonomy; (b) **non-directive art-making**, which supports competence through self-paced exploration; and (c) **non-verbal, sensory-rich activities**, which promote relatedness and emotional expression, particularly for children and adolescents with limited verbal communication. These elements are illustrated in Figure 1, which conceptualizes the relationship between art therapy and the fulfillment of psychological needs in children and adolescents with ASD.

**Freedom and Choice.** Art therapy provides a compelling avenue for fostering autonomy by offering children opportunities to make choices and exercise personal agency [32]. Within these therapeutic settings, children are encouraged to engage in art-making that reflects their individual preferences, interests, and emotional needs, free from rigid expectations or predetermined outcomes. This open-ended structure enables spontaneous, self-directed expression, particularly evident in multimodal arts activities, where children can choose among various mediums—such as painting, music, or movement—and transition between them as they wish [18].

Such autonomy-supportive environments not only enhance children’s sense of control but also contribute to feelings of competence, thereby promoting positive psychosocial and behavioral outcomes [25]. The inherently enjoyable and self-directed nature of art-making further facilitates relatedness, as children and therapists collaboratively explore the deeper meanings embedded in the creative process, fostering trust and emotional connection [18,32]. Moreover, completing a personally meaningful art product reinforces competence by empowering children to take ownership of the entire creative process—from inception to final realization—thereby strengthening their confidence and sense of achievement [18,32].

**Non-directive Arts-Making.** A defining feature of art therapy is its non-directive and facilitative approach, in which therapists adopt a supportive rather than prescriptive role [18,20]. Therapists honor each child’s pace and preferences, offering gentle guidance and constructive feedback that nurture both autonomy and competence. This respectful dynamic fosters a genuine therapeutic alliance, which may serve as a model for broader social engagement and connection [33].

In addition to supporting individual expression, collaborative arts-based projects—such as group murals or ensemble music-making—offer opportunities for children with ASD to practice and develop social relatedness [34,35]. The non-directive nature of these group activities encourages spontaneous social interaction, enabling children to engage in authentic communication and relationship-building within a relaxed, low-pressure environment. Such settings support the development of perspective-taking, empathy, and cooperation—skills that are often challenging for children on the spectrum [20,32,35,36].

**Non-verbal and Sensory-Rich Activities.** The non-verbal and sensory-rich nature of art therapy makes it particularly well-suited for children with ASD who experience sensory over-responsiveness and limited verbal communication abilities [37,38]. By engaging in the expressive and non-cognitive domains of the brain, arts-based activities provide alternative pathways for self-expression and interpersonal communication [20,32]. For example, in family therapy sessions, children may use movement, singing, or visual art to express affection—bypassing the need for verbal articulation and enabling more authentic emotional expression.

The sensory-rich qualities of art therapy—such as the tactile experience of sculpting, the visual stimulation of colors, and the auditory engagement of music—can support sensory integration and emotional regulation in children with ASD [20]. These structured therapeutic experiences help children become more familiar with sensory stimuli and learn to manage hypersensitive responses within a safe and supportive environment. Over time, such engagement may lead to improvements in social behavior, emotional flexibility, and adaptive functioning.

Moreover, effective sensory integration lays the foundation for emotional self-regulation, which is essential for developing social competence and behavioral self-management. As children become more confident in managing sensory input, they are more likely to experience a sense of autonomy and competence in navigating the often unpredictable and overstimulating social environment [39,40,41].

### 1.3. Research Gap and Research Questions

Despite growing interest in art therapy for children with ASD, the academic community continues to grapple with clarifying its overall effectiveness. Existing review studies frequently focus on single-case designs, isolated art modalities, or narrowly defined outcome categories, which limits the generalizability and applicability of findings [13,14,15,16,20,42]. Moreover, there is a lack of comprehensive synthesis evaluating both the methodological rigor and multidimensional outcomes of art therapy for children with ASD. To address this gap, the present study conducts a systematic review to assess the effectiveness and methodological quality of art therapy interventions for children with ASD. This study is guided by the following research questions:

1. What outcomes have been identified in art therapy for children and adolescents with ASD?
2. How effective is art therapy in promoting positive developmental outcomes in children and adolescents with ASD?
3. What theoretical frameworks have guided the design of art therapy for children and adolescents with ASD?
4. What intervention strategies—regarding art modality and format (e.g., individual vs. group)—are associated with positive outcomes?
5. What is the methodological rigor of existing art therapy studies for children and adolescents with ASD?

## 2. Methods

This systematic review was conducted in accordance with the PRISMA 2020 guidelines, ensuring transparency and methodological rigor [43]. This review was registered on the Open Science Framework (OSF). Registration DOI: https://doi.org/10.17605/OSF.IO/XAG58 (accessed on 1 November 2025).

### 2.1. Search Strategy

To evaluate the effectiveness of various forms of art therapy for children and adolescents with ASD, a comprehensive search was conducted across eight electronic databases: PsycINFO, PsycARTICLES, PubMed, Web of Science, Scopus, EMBASE, CINAHL, and Medline. The search was conducted with a cutoff date of 1 November 2024, and included only articles published prior to this date. A detailed list of search terms is provided in Appendix A.

### 2.2. Study Selection

All studies were manually screened and data were independently extracted by two reviewers. At each stage, each reviewer conducted the initial screening independently and resolved the discrepancies through subsequent discussion. Studies were included based on the following criteria: (a) randomized controlled trials (RCTs); (b) participants aged 18 years or younger diagnosed with ASD; (c) interventions employing art therapy approaches. In this study, art therapy refers to interventions using visual art, music, dance/movement, drama, poetry/writing, and intermodal or multimodal approaches as the primary intervention modality (See definitions in Appendix B); (d) sample size of more than 15 participants per experimental or control group to ensure adequate statistical power and reduce the risk of bias; (e) publication in peer-reviewed journals; and (f) written in English to ensure language consistency and facilitate access to a broad and internationally recognized body of scholarly work. Studies were excluded if they: (a) did not report quantitative results; or (b) focused on parents rather than children and adolescents.

### 2.3. Data Synthesis and Analysis

Information from each identified study was synthesized based on study background, participant characteristics, research aims or hypotheses, intervention details, outcomes, and significance of outcome variables. Data were extracted manually without the guidance of formal guidelines. Two independent reviewers conducted data extraction and cross-checking to ensure accuracy, and regular meetings were held to discuss study progress and emerging findings.

### 2.4. Assessment of Methodological Rigor

To assess methodological rigor, two separate rating scales were employed. The first was the Cochrane Risk of Bias tool (ROB 2.0) [44], and the second was the Delphi List [45], supplemented with four additional self-designed items. In this review, the score of methodological rigor will not be considered as an inclusion criterion. To ensure inter-rater reliability, two independent reviewers conducted the rating process using both scales.

#### 2.4.1. Cochrane Risk of Bias Tool (ROB 2.0)

The ROB 2.0 tool is a widely recognized instrument designed to assist reviewers in assessing the risk of bias in RCTs due to methodological shortcomings. It includes 22 structured items grouped into five domains: (1) bias arising from the randomization process, (2) bias due to deviations from intended interventions, (3) bias due to missing outcome data, (4) bias in outcome measurement, and (5) bias in the selection of the reported result. Each item is evaluated using one of five response options: Yes, Probably yes, Probably no, No, or No information. Based on these responses, each domain is rated as having a low risk, some concerns, or high risk of bias. A low-risk rating indicates strong confidence that the study results are unlikely to be affected by bias. A rating of some concerns reflects uncertainty regarding the validity of the findings due to potential methodological limitations. A high-risk rating suggests serious methodological flaws in at least one domain, which are likely to substantially compromise the credibility of the study outcomes [44].

#### 2.4.2. Delphi List with Four Additional Items

To complement the ROB 2.0 tool, the Delphi List [45] was also employed to assess the methodological quality of the included studies. The Delphi List is a widely accepted instrument for appraising the quality of RCTs and consists of nine items. The assessed criteria include: (1) reporting of randomization, (2) allocation concealment, (3) baseline comparability, (4) specification of eligibility criteria, (5) blinding of participants, (6) blinding of service providers, (7) blinding of outcome assessors, (8) reporting of point estimates and measures of variability, and (9) use of intention-to-treat analysis [45]. To enhance the comprehensiveness of the quality assessment, four additional items were incorporated to reflect contemporary methodological standards, based on the Consolidated Standards of Reporting Trials (CONSORT) guidelines [46]. The assessed criteria include: (10) Sample size justification and power calculation, (11) Follow-up sufficiency and reporting, (12) Trial registration, (13) Statistical appropriateness. The complete list of quality assessment items is presented in Appendix C. Overall, the 13 items encompass four key domains of methodological quality: (i) research design (item 1, 2, 3, 5, 6, 7, 9), (ii) participant recruitment (item 4, 10), (iii) intervention protocol and follow-up (item 11, 12), and (iv) statistical appropriateness and outcome reporting (item 8, 13).

The 13 items was evaluated using a dichotomous scoring system (Yes = 1; No/Unclear = 0), resulting in a maximum possible score of 13 points. Higher total scores indicated greater methodological quality. As the Delphi List does not specify a cut-off score, tertile-based classification was applied to align with the three-level evaluation framework of the ROB 2.0 tool. Specifically, scores above 8 (i.e., >66.6% of the maximum) were categorized as high methodological quality; scores between 5 and 8 were considered moderate quality; and scores below 5 (i.e., <33.3% of the maximum) were classified as low quality.

## 3. Results

**Figure 2:**
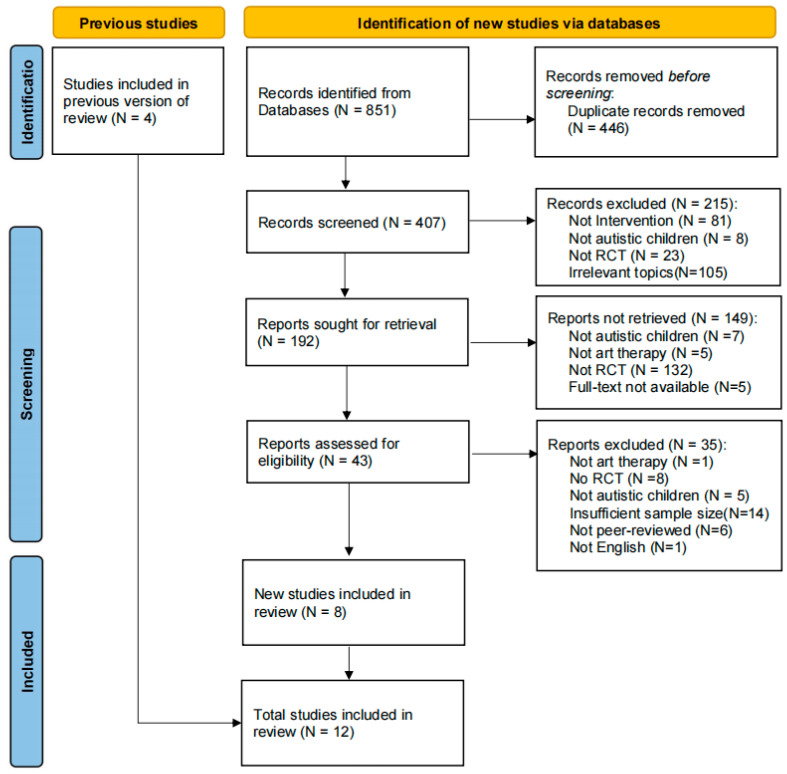
Flow diagram for systematic reviews which included searches of databases.

As shown in the PRISMA flow diagram (Figure 2), the initial search yielded a total of 851 articles across eight electronic databases. Both reviewers conducted an initial screening based on titles and abstracts, removing duplicates and ineligible records. Following this screening, 43 articles remained for full-text review. Additionally, four systematic reviews were screened to supplement the search [13,14,15,16], resulting in four additional articles being included. After a thorough full-text review, 12 articles were selected, which were published between 2017 and 2024 and encompassed a total of 899 participants aged 5 to 11 years diagnosed with ASD (see Table 1).

The studies demonstrated broad international representation: three were conducted in China [49,50,53], two in the United States [48,52], and two in Iran [56,57]. Additional studies were conducted in Iraq [51], France [55], and Canada [58]. Two studies included participants from multiple countries [47,54].

### 3.1. Effectiveness of Art Therapy

**Overall ASD Symptoms.** Among the 12 studies included in this review, three evaluated changes in overall ASD symptoms [50,53,55]. These studies assessed all core domains of ASD, including impairments in social communication and interaction, repetitive behaviors, and atypical sensory processing. Standardized assessment tools were employed, such as the Autism Diagnostic Observation Schedule [59], the Autism Behavior Checklist [60], the Childhood Autism Rating Scale [61], and the Clinical Global Impression scale [62]. All three studies reported a statistically significant reduction in overall ASD symptom scores among participants in the experimental groups compared to those in the control groups.

**Social communication and social interaction.** Among the 12 studies reviewed, seven reported outcomes related to social communication and interaction [47,48,52,53,54,56,58]. These outcomes were assessed using standardized instruments, including the Social Responsiveness Scale [63], Peer Interaction Paradigm [64], Matson Social Skills Questionnaire [65], and the Children’s Communication Checklist—Second Edition [66]. Of the seven studies, five reported statistically significant improvements in social communication and interaction following arts-based interventions in the experimental groups [48,52,53,56,58], while two studies observed improvements that were not statistically significant [47,54]. Collectively, these findings suggest that arts-based interventions may positively influence social communication skills in children and adolescents with ASD.

**Psychological outcomes.** Among the 12 studies reviewed, two reported psychological outcomes [49,52], specifically stress-related symptoms such as perceived pressure [49] and anxiety [52]. Ding [49] found significant reductions in perceived pressure following art therapy. Ioannou et al. [52] observed notable improvements in trait anxiety—defined as a general tendency to experience anxiety—over time. However, no significant changes were reported in state anxiety, which refers to anxiety triggered by specific situational stressors [67]. Overall, these findings suggest that art therapy may be effective in alleviating general stress-related symptoms in children and adolescents with ASD.

**Behavioral outcomes.** Of the 12 studies reviewed, two reported behavioral outcomes, specifically sleep habits and maladaptive behaviors [49,58]. These outcomes were assessed using the Children’s Sleep Habits Questionnaire [68] and the Maladaptive Behavior Subscale of the Vineland Adaptive Behavior Scales [69]. Ding [49] reported significant improvements in sleep habits following art therapy, whereas Sharda et al. [58] found no significant changes in maladaptive behaviors. Overall, the evidence regarding the effectiveness of art therapy in improving behavioral outcomes remains limited and warrants further investigation.

**Other outcomes.** Other outcomes reported in the reviewed studies included motor skills [51,57], neurologically related development [53,58], and language ability [47,53,58]. Two studies evaluated motor skills [51,57], both utilizing the Lincoln–Oseretsky Motor Development Scale [70]. Imankhah et al. [51] reported significant improvements in overall motor skills, and Sabet and Abadi [57] observed notable enhancements in fine motor skills, balance, and physical flexibility. These findings suggest that arts-based therapies may be effective in enhancing motor functioning in children and adolescents with ASD.

Two studies assessed neurologically related development [53,58]. Liu et al. [53] reported that participants in the experimental group demonstrated significantly greater improvements in neural development scores, as measured by the Gesell Developmental Schedules [71]. Similarly, Sharda et al. [58] observed significant enhancements in functional brain connectivity using resting-state functional magnetic resonance imaging (rs-fMRI). These findings provide preliminary evidence that art therapy may promote positive neurological development in children and adolescents with ASD.

Three studies assessed language-related outcomes [48,53,58]. Sharda et al. [58] found no significant changes in receptive vocabulary, as measured by the Peabody Picture Vocabulary Test, Fourth Edition [72]. In contrast, Corbett et al. [48] reported significant improvements in verbal ability, assessed using the Theory of Mind–Verbal Interactions test. Similarly, Liu et al. [53] observed improvements in language ability, measured by the Psycho-Educational Profile, Third Edition [73].

### 3.2. Intervention Format

**Arts modalities.** The reviewed studies employed various arts modalities as intervention approaches, including music, drama, painting, and multimodal arts. Among the 12 studies, six utilized music-based interventions [47,50,53,54,55,58].

Of these, five incorporated improvised activities such as singing, dancing, playing musical instruments, joint musical engagement with a therapist (e.g., singing or instrumental play), improvisational performance, instrument selection, personalized music listening sessions, and both instrumental and vocal improvisation [47,50,54,55,58]. One study applied the TOMATIS method, which targets neurosensory stimulation through auditory training [53].

Four studies employed drama-based interventions [48,49,52,56]. These interventions included theater games, role-playing exercises, protagonist character development, puppet shows, small group activities, and live performances [48,49,52,56].

Additionally, one study utilized an intermodal arts approach, combining music with body movement [51]. Another study implemented a visual arts-based intervention, incorporating activities such as painting, paper tearing, pottery, and handicraft making [57].

In terms of modality-specific effectiveness, music therapy demonstrated efficacy in improving core ASD symptoms [50,53,55], as well as outcomes related to social communication and interaction, language ability, and neurological development [53,58]. Drama therapy was effective in enhancing social communication and interaction [48,52,56], reducing anxiety [48,49,52], improving behavioral regulation [50], and supporting language development [48]. The single study employing painting therapy reported improvements in fine motor skills, balance, and flexibility [57]. Additionally, the study utilizing a mixed-modality approach combining music therapy with dance and movement also demonstrated effectiveness in enhancing motor skills [51].

**Group versus one-on-one format.** Six studies employed a group-based intervention format [48,49,51,52,55,56], including four that utilized drama therapy [48,49,52,56], one used music therapy [55], and one that combined music therapy with dance and movement [51]. Six studies adopted an individual (one-on-one) format [47,50,53,54,57,58], comprising five that implemented music therapy [47,50,53,54,58] and one that used painting therapy [57]. 

Overall, individual therapy was more frequently applied in music-based interventions. Group therapy was predominantly used in drama-based interventions.

**Duration and frequency.** The majority of the reviewed studies (N = 8) had intervention durations of three months or less [48,49,50,51,52,56,57,58], while interventions were conducted once a week [48,52,55,56,58], twice a week [51], or three or more times per week [53,54].

### 3.3. Theoretical Underpinnings Guiding Intervention

Among the 12 reviewed studies, five explicitly referenced theoretical frameworks [48,49,50,53,58], while seven did not discuss their theoretical underpinnings [47,51,52,54,55,56,57]. Sharda et al. [58] employed a reward-based cortical modulation and sensorimotor integration framework to guide the design of music therapy. Two studies cited auditory integration theory [50,53] as the basis for music therapy interventions. Social learning theory [48] and analytical psychology [49] were used to offer grounding for drama therapy.

### 3.4. Methodological Rigor

The Cohen’s kappa value for the assessment of methodological rigor was 0.803, indicating a high level of inter-rater reliability.

#### 3.4.1. Risk of Bias

**Figure 3:**
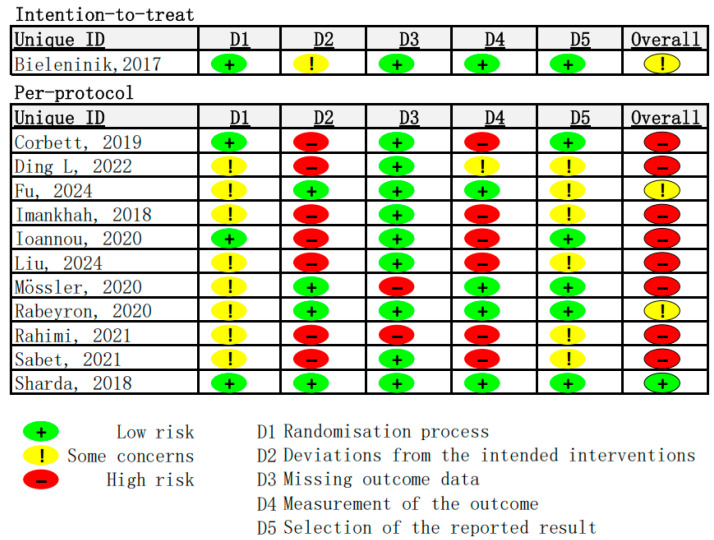
Results of risk of bias assessment (N = 12) [47,48,49,50,51,52,53,54,55,56,57,58].

Risk of bias was assessed for all included studies using the ROB 2.0 tool (see Figure 3). Of the 12 studies reviewed, only one employed an intention-to-treat (ITT) analysis [47], which includes all participants as originally allocated after randomization, regardless of adherence to the intervention protocol. The remaining 11 studies utilized per-protocol (PP) analysis, which includes only participants who completed the study in accordance with the protocol [74]. The study that used ITT was rated as having “some concerns” [47]. Among the 11 studies that employed PP analysis, eight were assessed as having a high overall risk of bias [48,49,51,52,53,54,56,57], two studies were rated as having “some concerns [50,55]” and one study was rated as having a “low risk of bias [58]”. Overall, the included studies exhibited a relatively high risk of bias, highlighting the need for cautious interpretation of their findings within the context of this systematic review.

#### 3.4.2. Methodological Quality

The methodological quality of the included studies was assessed using a 13-item checklist comprising the Delphi List [45], supplemented with four additional items (see Appendix C). The mean quality score was 5.92 (*SD* = 2.72), with a median score of 7, out of a maximum possible score of 13. Based on the predefined cut-off criteria, two studies scored above the 66.6th percentile (≥9), indicating high methodological quality. Six studies scored between 5 and 8, representing moderate quality, while four studies fell within the lowest 33.3rd percentile (≤4), indicating low methodological quality (see Appendix D).

**(i) Research design.** All the studies included were RCTs. Allocation concealment during the randomization process was reported in five studies [47,48,52,54,58], typically involving third-party procedures, while the remaining seven did not provide sufficient information regarding this aspect. Baseline comparability was confirmed in seven studies [47,48,50,52,53,54,58], supporting the validity of the randomization process; however, five studies did not report baseline equivalence. Regarding blinding procedures, two studies reported that participants were blinded to group allocation [50,55]. None of the studies reported blinding of intervention providers, and four studies indicated that outcome assessors were blinded [47,50,54,58].

**(ii) Participants recruitment.** Most of the included studies (N = 11) clearly defined and operationalized eligibility criteria for participation in the intervention. However, in terms of sample size, 11 studies recruited participants that were fewer than the recommended minimum of 64 per group, except for Bieleninik et al. (2017) [47]. Furthermore, only two studies reported a formal sample size justification [50,55].

**(iii) Intervention protocol and follow-up.** Regarding intervention protocols, six studies reported clinical trial registration [47,48,52,54,55,58], while the remaining studies did not provide any information on this aspect. In terms of follow-up procedures, only three studies conducted follow-up assessments and reported corresponding results [47,54,56].

**(iv) Statistical appropriateness and outcome reporting.** In terms of statistical analysis, four studies utilized statistically inappropriate approaches, such as independent t-tests and one-way ANCOVA [49,51,53,57]. Most of the included studies (N = 10) reported outcome variability, typically presenting results with means and standard deviations. Two studies did not report these descriptive statistics [49,53].

## 4. Discussion

This study evaluated the effectiveness of art therapy for children and adolescents with ASD and reviewed the implementation strategies and methodological quality of existing intervention studies. A total of 12 RCTs were included, encompassing various forms of art therapy: music therapy (N = 6), drama therapy (N = 4), painting therapy (N = 1), and therapy using mixed modalities (N = 1). All studies included at least 15 participants per group. The findings of this systematic review suggest that art therapy shows promise in reducing overall ASD symptoms and stress-related psychological outcomes, as well as in enhancing social communication, motor skills, language ability, and neurodevelopmental functioning in children with ASD. However, the review also identified a generally high risk of bias and low methodological rigor among the included studies.

A key contribution of this study is that, among existing reviews on the effectiveness of art therapy for children and adolescents with ASD, it is the first systematic review to include only RCTs with adequate sample sizes, thereby providing more reliable and generalizable findings. In addition, the study offers a thorough and systematic evaluation of the methodological rigor of current RCTs, including assessment of risk of bias, intervention dosage, follow-up procedures, and statistical analyses, and highlights important directions for future research.

### 4.1. Effectiveness of Art Therapy for Children and Adolescents with ASD

Regarding outcome variables, this review found that social communication and interaction was the most frequently reported outcome among the included studies [47,48,52,53,54,56,58]. Other commonly reported outcomes included overall ASD symptoms [50,53,55], language ability [48,53,58], stress-related psychological outcomes [50,61] and behavioral outcomes [49,58], motor skills [51,57], and neurologically related development [53,58].

In terms of the effectiveness of art therapy for children and adolescents with ASD, results of this systematic review revealed that, among the studies reporting the outcomes above, more than half demonstrated significant improvements in social communication and interaction (5 out of 7, 5/7), overall ASD symptoms(3/3), anxiety/stress reduction (2/2), motor skills (2/2), language ability (2/3), and neurologically related development (2/2), suggesting a positive effect of art therapy in these domains. However, evidence regarding behavioral outcomes—including sleep habits (1/1) and maladaptive behaviors (0/1)—remains inconclusive.

The current findings align with several previous reviews on the effectiveness of art therapy for individuals with ASD. For example, Bololia et al. [14] reported that dramatherapy can foster social skills and promote emotional well-being, and Vogel et al. [16] observed improvements in both social and motor skills. Notably, although there are some consistent findings across certain variables, the number of RCTs examining these outcomes remains relatively small, and the positive results may, in part, be influenced by publication bias. Therefore, more empirical studies are needed to further verify the effectiveness of art therapy.

### 4.2. Theoretical Frameworks Have Guided the Design of Art Therapy

Among the 12 reviewed studies, only five explicitly referenced theoretical frameworks [48,49,50,53,58]. Of these five studies, three were music therapy interventions, all of which mentioned auditory integration theory as their theoretical basis [50,53,58]. The remaining two studies were drama therapy, which drew on social learning theory [48] and analytical psychology [49] to provide a theoretical foundation for their interventions.

These results reveal a trend toward using auditory integration theory to explain the effects of music therapy, with intervention designs emphasizing sensory processing and auditory experience. In addition, this result also shows that intervention designs based on behavioral theory and psychoanalytic theory emphasize social learning and social participation, reinforcing the imitation of social behaviors and the development of mastery in social experiences. Analytical psychology offers a holistic approach that supports the integration of the unconscious and conscious self with the external world and releases emotional tension on an unconscious level within a safe therapeutic context.

However, it can also be observed that current studies lack emphasis on humanistic and positive psychology theories, even though art therapy inherently highlights client-centered and strength-based practice. Based on this, we propose incorporating SDT into the theoretical frameworks of future studies. The arts-based therapies reviewed in this study draw upon various theoretical frameworks aligned with SDT [25,26,27,28], for example, creating a therapeutic environment that encouraged participants to explore personal preferences and share personal experiences to connect their inner world with the external world through artwork [53]. This approach supports the SDT principles of autonomy and relatedness. Similarly, drama therapy promotes interpersonal connection and competence development through peer interaction and performance-based activities. These elements resonate with SDT’s emphasis on intrinsic motivation and psychological growth. By engaging participants in expressive modalities that affirm their agency and sense of social belonging, these therapies exemplify how SDT can be applied within creative mental health interventions.

Furthermore, this result also reveals a considerable inconsistency in the theoretical foundations of current art therapy research. Since most studies (7/12) did not explain the theoretical basis of their intervention design, we strongly recommend that future research pay greater attention to this aspect.

### 4.3. Intervention Formats of Art Therapy for Children and Adolescents with ASD

Regarding the format and modality of interventions, music therapy (N = 6) and drama therapy (N = 4) were the most frequently employed approaches. Music-based interventions incorporated improvised activities such as singing, dancing, playing musical instruments, joint musical engagement with a therapist, improvisational performance, and personalized music listening sessions. In contrast, drama-based interventions included components such as role-playing, rehearsals, and performance, which offer specific therapeutic benefits by actively engaging participants in social scenarios. An interesting finding of this study is that music therapy reported more benefits for overall ASD symptoms [50,55], while drama therapy showed promising effects on social skills [48,52,56]. Although this study cannot examine this in depth, considering the differences in activities and theoretical foundations, it is worthwhile to explore whether music therapy and drama therapy benefit individuals with ASD in different aspects.

Six studies utilized individual interventions, while six implemented group-based formats. Individual therapy was more frequently applied in music-based interventions, whereas group therapy was predominantly used in drama-based interventions.

The majority of the reviewed studies had intervention durations of less than three months (N = 8), and majority of sessions were conducted two or less a week (N = 6). Compared with the recommendations of Brentani et al. [9], which suggest intensive treatment—five days a week for a minimum of five hours per day, the interventions included in the current review appear to be low in dosage.

### 4.4. Methodological Rigor of Existing Art Therapy Studies for Children and Adolescents with ASD

Across the reviewed studies, a high risk of bias and generally low methodological rigor were identified. The results of ROB 2.0 show that most studies (N = 8) were rated as having a high overall risk of bias, suggesting serious flaws in study design. One major limitation is that, although all studies employed RCT designs, most relied on PP analyses rather than ITT approaches, introducing potential bias by excluding participants who deviated from the study protocol—PP analyses often exclude participants who drop out or deviate from the protocol, removing less favorable outcomes and potentially inflating treatment effects [74]. When combined with publication bias, which favors the reporting of positive results [75,76], the literature may overestimate the true effectiveness of art therapy. This convergence of biases highlights the need for future studies to employ ITT analyses and preregistered protocols to ensure more accurate and transparent evaluations of therapeutic outcomes [77].

Another major limitation was the inadequate sample size. The majority of the included studies (N = 8) enrolled between 15 and 30 participants per group, which falls below the recommended sample size of 64 per group for adequate statistical power, raising concerns about the increased risk of Type II errors. As a result, some studies might have failed to detect significant effects not because the interventions were ineffective, but rather due to insufficient statistical power.

Other limitations on methodology include the randomization process, blinding procedures, trial registration, and sample size calculations—were largely overlooked among the included studies. These limitations collectively reduce confidence in the reported findings and underscore the need for more rigorously designed trials in future research.

### 4.5. Implications for Research and Practice

This systematic review has several important implications for both future research and clinical practice regarding art therapy on ASD. First of all, while art therapy demonstrates potential as a complementary approach for promoting positive outcomes in children with ASD, the current evidence base is constrained by low methodological rigor. Many studies rely on small sample sizes, increasing the likelihood of Type II errors, and frequently employ PP analyses rather than ITT approaches, despite utilizing RCT designs. This review provides a strong emphasis on the necessity of robust methodological practices to enhance confidence in reported outcomes. Generating evidence-based insights is essential for the development and broader application of arts therapies for neurodiverse populations, thereby advancing the field of applied psychology through innovative and inclusive approaches.

Secondly, a promising direction for future research involves the integration of technology to more precisely measure therapeutic change. The present review found that most studies relied on self-report measures. Due to the client-centered and improvisational nature of art therapy and the specific characteristics of the ASD population, conducting large-scale quantitative studies may be challenging. One possible direction is to understand neural changes during art therapy. Notably, we identified one study that employed neuroimaging techniques and demonstrated enhanced brain connectivity following music therapy [58]. Future research could further explore the potential impact of art therapy on brain development.

Thirdly, to establish best practices, further implementation studies are needed to identify components that may influence the effectiveness of art therapy—such as optimal session frequency, modality selection, therapeutic settings, and ASD severity. Future research could also investigate hypothesized process variables grounded in SDT [26,27], including autonomy, relatedness, and competence.

From a practical standpoint, practitioners are encouraged to design intervention sessions informed by theoretical frameworks and structured formats, incorporating diverse art modalities and considering the appropriateness of group versus individual settings. Given the unique challenges associated with working with neurodiverse children, facilitators must remain attuned to their individual needs and preferences. Rather than viewing these children through a deficit-based lens, a more constructive approach involves creating autonomous, creative, and enjoyable environments—such as those offered by art therapy—that affirm their identities and foster meaningful engagement.

### 4.6. Limitations

The findings of this study should be interpreted in light of several limitations. First, only 12 studies met the inclusion criteria, which limits the available evidence regarding the effectiveness of art therapy in improving various outcomes.

Second, due to the limited number of studies and difficulties in obtaining datasets, despite efforts to contact authors, the available data were ultimately insufficient to conduct a convinced meta-analysis (only 4 studies for social communication outcomes and 3 for overall ASD symptoms). Consequently, the conclusions of this review are based on qualitative synthesis and cannot indicate statistical significance and may be influenced by the researchers’ training and interpretive stance.

Third, the exclusion of non-English publications may have inadvertently omitted relevant research, limiting the representativeness and cultural diversity of the findings. Furthermore, the exclusion of interventions targeting caregivers limited the review’s ability to assess the effectiveness of family-involved interventions, which may represent an important component of therapy [9].

Fourth, during the article screening process, reviewers conducted initial screening at each stage and resolved discrepancies through discussion. Therefore, inter-rater reliability for the entire screening process is not available. Future research could implement independent screening procedures and report inter-rater reliability to enhance methodological rigor and ensure objectivity.

## 5. Conclusions

This systematic review synthesized evidence from 12 RCTs to evaluate the effectiveness of art therapy for children and adolescents with ASD. The findings indicate that art therapy, particularly music and drama therapy, can enhance social communication, language ability, motor skills, and neurodevelopment, while also reducing stress-related symptoms and overall ASD severity. However, most studies demonstrated small sample sizes, brief intervention durations, and low methodological rigor, limiting the generalizability of current evidence. Only a few studies reported theoretical underpinnings, revealing a lack of consistent frameworks to guide intervention design. Future research should prioritize methodological improvements, such as larger sample sizes, trial registration, intention-to-treat analyses, and longitudinal follow-ups, to strengthen the evidence base. Moreover, incorporating theoretical perspectives like SDT could better explain the mechanisms of change and emphasize autonomy, competence, and relatedness within creative interventions. Overall, art therapy represents a promising, person-centered, and holistic approach for supporting the developmental and psychological needs of children and adolescents with ASD, but further rigorously designed studies are required to establish its efficacy and inform evidence-based practice.

## Figures and Tables

**Table 1:** Characteristics of included studies.

Study Background			Participants			Aims	Intervention					Outcomes						
ID	Author (Year)	Region (s)	EXP *n* (% M)	CTRL *n* (% M)	Mean Age		Arts Modality + Components in EXP Group	Sessions	Length	Group vs. Individual	Theory	ASD Sx. (Overall)	Social	Psych.	Beh.	Physical	Language	Neuron Dev.
1	Bieleninik, 2017 [47]	Multisite	182 (94%)	182 (81.9%)	5.5	Evaluate the effectiveness of improvisational music therapy in general social communication skills of children with ASD.	Improvisational music therapy vs. Enhanced standard care (ESC) Joint singing or musical instruments playing, and use of improvisation techniques such as synchronizing, mirroring, or grounding.	60 sessions (high intensity) 30 sessions (Low IMT)	5 mths	Individual led by qualified music therapist	NA		✘ (ADOS-Social Affect; SRS)					
2	Corbett, 2019 ^a^ [48]	USA	44 (66.7%)	33 (73.1%)	10.9	Evaluate the effectiveness of treatment in cooperative play and verbal interaction during peer interaction in children with ASD.	SENSE Theater vs. Waitlist control (WLC) Theater games, role playing, improvisation, and character development, while putting on a final play.	10	10 weeks	Peer led Group	Social Learning Theory		✔ (Sig. in Cooperative Play; PIP)				✔ (Sig. in verbal ability; ToM-Verbal Interactions)	
3	Ding, 2022 [49]	China	30 (NA)	30 (NA)	--	No Information. (Examine effectiveness of drama therapy in sleep quality and mental health in children with ASD.)	Drama Therapy vs. Treatment as usual (TAU) Drama performance.	NA	3 mths	Group	Analytical psychology	--	--	✔ (Sig, in Perceived pressure; CPSS)	✔ (Sig. in Sleeping habit; CSHQ)			
4	Fu, 2024 [50]	China	15 (86.6%)	15 (80%)	6.9	Examine effectiveness of TOMATIS training in improving autism- related symptoms in children with ASD.	TOMATIS vs. Music Listening Only Listening to 4 different pieces of music 1–2 times a day for 1–2 h with sound settings of different frequencies, strength of air conduction, bone conduction, and the processing time delay.	2 (Each session lasted 10 days)	27 days, divided into two 10 days sessions with 7 rest days	Individual led by TOMATIS trainers	Auditory Integration Theory	✔ (CARS; ABC)						
5	Imankhah, 2018 [51]	Iraq	15 (100%)	15 (100%)	6–11	Explore the effect of music therapy along with physical activity on motor coordination behaviors of children with ASD.	Multimodality (Music therapy and movement) vs. Blank Control Using images for contact, eye contact, singing familiar songs, teaching rhythm through body movements, and engaging in balance board games and rhythmic motor games.	15	7.5 week	Group led by Psychologists	NA	--	--			✔ (Sig. in Motor Skills; LOMST)		
6	Ioannou, 2020 [52]	USA	44 (66.7%)	33 (73.1%)	10.8	Investigate the effectiveness of SENSE Theatre in enhancing social play and decreasing anxiety levels.	SENSE Theater vs. WLC Mock auditions, theater games, imaginative play, character development, role-play, and rehearsals; Video modeling for home practice; Public performances	10 four-hour sessions (Daily)	10 weeks	Group; with Trained peers	NA		✔ (Sig. in group and self-play; PIP)	✔ (Sig. In trait anxiety) ✘ (Non-Sig in state anxiety; STAI-C)				
7	Liu, 2024 [53]	China	40 (70%)	40 (62.5%)	5.58	Examine the effectiveness of Music Therapy with Auditory Integration Training (AIT) in the overall development of children with ASD.	Music Therapy + AIT vs. AIT only MT: Improvisational Performance, Instrument Selection, Instrumental Performance, and activities with peers. AIT: Oral training with pinyin imitation, vocalization using animated feedback, vocalization training, perceptual coordination by adjusting pitch, memory training by recalling previously learned sounds and interpreting silent animations.	72	6 mths	Individual; led by music therapist	Auditory Integration Theory	✔ (ABC)	✔ (ATEC)				✔ (Sig in. Language ability; PEP-3)	✔ (GDS)
8	Mössler, 2020 [54]	Multisite	50 (84%)	51 (84%)	5.4	Examine the effectiveness of musical and emotional attunement in improving core ASD traits in children with ASD; and the relationship between therapy intensity and the level of attunement achieved.	Improvisational Music Therapy (High Intensity vs. Low intensity) Joint singing or musical instruments playing, and use of improvisation techniques such as synchronizing, mirroring, or grounding.	High intensity (max. 60 sessions) Low intensity (20 sessions)	5 mths	Individual; led by qualified music therapist	NA		✘ (AQR)	--				
9	Rabeyron, 2020 [55]	France	19 (87%)	18 (87%)	7.9 (0.79)	Examine the effectiveness of music therapy in improving clinical ASD symptoms in children with ASD.	Music Therapy vs. Music listening Only Playing instrumental and vocal music, Instrumental and vocal improvisation.	25 (30 min per session)	8 mths	Group; led by a music therapist, a co-therapist and clinical psychology trainee.	NA	✔ (CGI; ABC) ✘ (CARS)	--	--				
10	Rahimi, 2021 ^b^ [56]	Iran	20 (NA)	20 (NA)	10.03 (3.14)	Examine the effectiveness of drama therapy in enhancing the social skills dimensions of children with ASD.	Drama Therapy vs. TAU Stretching, memory exercises, group games, and five senses exercises, puppet shows, behavioral& participatory training, and animal play. Appling learned skills in previous sessions for social interactions.	12	12 weeks	Group; Led by trained researchers & therapeutic assistants.	NA	--	✔ (MSSQ)					
11	Sabet, 2021 [57]	Iran	15 (NA)	15 (NA)	6–12	Examine whether painting-based art therapy can improve fine and gross motor skills among Children with ASD.	Painting-based art therapy vs. TAU Painting with contrasting colors, tearing and crumpling paper, working with pottery, making handicrafts, cutting lines with scissors, and purposeful coloring.	18	6 weeks	Individual; led by Psychologist	NA	--	--			✔ (Sig. in fine motor skills, balance; flexibility; LOMST)		
12	Sharda, 2018 [58]	Canada	26 (80.8%)	25 (88%)	10.30 (1.91)	Investigate the effectiveness of a music-based intervention in improving social communication, and functional brain connectivity in school-age children with ASD.	Music Therapy vs. Treatment as usual Musical instruments, songs, and rhythmic cues to enhance communication, turn-taking, sensorimotor integration, social appropriateness, and musical interaction.	8–12	8–12 weeks	Individual; led by accredited music therapist	Reward-based cortical modulation and sensoria-motor integration.	--	✔ (Sig. in prag. communication; CCC-2) ✘ (Non-sig. in social responsiveness; SRS)		✘ (Non-sig. in mal-adaptive behavior; VABS)		✘ (Non-sig. in vocab. reception; PPVT-4)	✔ (Sig. in increasing brain connectivity)

Notes: ABC = Autism Behavior Scale; ADOS = Autism Diagnostic Observation Schedule; ATEC = Autism Treatment Evaluation Checklist; CARS = Childhood Autism Rating Scale; AQR = Assessment of the Quality of Relationship; CCC-2 = Children’s Communication Checklist; CGI = Clinical Global Impression; CPSS = Pressure Perception Scale (Chinese version); CSHQ = Children’s Sleep Habits Questionnaire; GDS = Gesell Developmental Schedules; LOMST = Lincoln-Oseretsky’s Motor Development Scale; MSSQ = Matson Social Skills Questionnaire; PIP = The Peer Interaction Paradigm; PPVT-4 = Peabody Picture Vocabulary Test; SRS = Social Responsiveness Scale; STAIC = State-Trait Anxiety Inventory for Children; TOM-Verbal/Contextual = Theory of Mind-Verbal/Contextual task; VABS-MB = Maladaptive Behaviour Subscale of the Vineland Adaptive Behaviour Scales. a. The sample overlapped with study of Ioannou [52]. b. According to the information provided by the corresponding author, outcome measurements of self-esteem and aggression were unclear, as such, we only looked at the aggregate social skills scores calculated by MSSQ.

## Data Availability

No new data were created or analyzed in this study. This review was registered on the Open Science Framework (OSF). Registration DOI: https://doi.org/10.17605/OSF.IO/XAG58 (accessed on 1 November 2025).

## Appendix A. Search Terms

(“expressive arts therapy” OR “expressive art therapy” OR “creative arts therapy” OR “multimedia arts therapy” OR “multimedia art therapy” OR “music therapy” OR “drama therapy” OR “dance therapy” OR “dance and movement therapy” OR “behavioral music therapy” OR “art therapy” OR “arts therapy” OR “theater education” OR “drama education” OR “visual art therapy” OR “visual arts therapy” OR “fine art therapy” OR “fine arts therapy”)

AND

(“intervention” OR “reduction” OR “management” OR “managing” OR “coping” OR “program” OR “programme” OR “training” OR “skills” OR “techniques” OR “counseling” OR “development” OR “modification” OR “modifying” OR “treatment”)

AND

(“autistic children” OR “autism” OR “ASD” OR “Autistic Spectrum Disorder” OR “autistic student” OR “developmental disorder” OR “autism spectrum disorder” OR “Asperger”)

AND

(“RCT” OR “randomized control trial” OR “randomized controlled trial” OR “randomized clinical trial” OR “randomised control trial” OR “randomised clinical trial” OR “randomised controlled trial”) [Abstract]

NOT

(protocol OR pilot) [Title]

AND

(filtered by peer-reviewed journal, human, English)

## Appendix B. Definitions of Different Types of Art Therapy

**Table A1:** Definitions of Different Types of Art Therapy.

Terms	Definition
Art therapy	Art therapy is a holistic therapeutic approach that integrates principles from mental health, human development, and psychological theory with various art modalities—such as visual art, music, movement, drama, and creative writing—to promote individual and community well-being [18].
Visual art therapy	Visual art therapy is defined as a “therapeutic process based on spontaneous or prompted creative expression using various art materials and art techniques such as painting, drawing, sculpture, clay modeling and collage” [78].
Music therapy	Music Therapy is the clinical & evidence-based use of music interventions to accomplish individualized goals within a therapeutic relationship by a credentialed professional who has completed an approved music therapy program [79].
Dance/movement therapy	The psychotherapeutic use of dance, movement, body awareness, and embodied communication to foster healing and well-being for all individuals, families, and communities [80].
Drama therapy	Drama therapy is one form of art therapy, which utilizes methods and techniques from the performing arts with principles of psychotherapy that promote transformation and evolution. Drama therapists employ a range of artistic techniques through methods such as storytelling, Greek myths, play scripts, puppetry, masks, and improvisation [81].
Poetry/writing therapy	Poetry therapy is the use of language, symbol, and story in therapeutic, educational, growth, and community-building capacities. It relies upon the use of poems, stories, song lyrics, imagery, and metaphor to facilitate personal growth, healing, and greater self-awareness. Bibliotherapy, narrative, journal writing, metaphor, storytelling, and ritual are all within the realm of poetry therapy [82].
Intermodal or Multimodal approaches	Intermodal expressive arts therapy involves the intentional use and integration of multiple artistic modalities within a therapeutic or growth-oriented process. These modalities may include visual arts (such as painting, photography, and crafts), movement and dance, voice, rhythm, sound, and music, as well as drama, enactment, and various forms of writing including poetry and storytelling. Additionally, intermodal practice may incorporate guided meditation, imaginative processes, and nature-based activities to facilitate self-expression, healing, and transformation [83].

## Appendix C. List of Quality Rating Items

**Table A2:** List of Quality Rating Items (The Delphi List and 4 Additional Items).

Items	Yes	No	Don’t Know
1. Was a method of randomization performed?	□	□	□
2. Was the treatment allocation concealed?	□	□	□
3. Were the groups similar at baseline regarding the most important prognostic indicators?	□	□	□
4. Were the eligibility criteria specified?	□	□	□
5. Was the outcome assessor blinded?	□	□	□
6. Was the care provider blinded?	□	□	□
7. Was the clients blinded?	□	□	□
8. Were point estimates and measures of variability presented for the primary outcome measures?	□	□	□
9. Did the analysis include an intention-to-treat analysis?	□	□	□
10. Was a sample size justification or power calculation provided?	□	□	□
11. Was the follow-up period sufficiently long (i.e., >3 months) and adequately reported?	□	□	□
12. Was the trial registered?	□	□	□
13. Were the statistical analysis methods clearly described, justified, and appropriate?	□	□	□

## Appendix D. The Results of Quality Rating Scale

**Table A3:** The Results of Quality Rating Scale (The Delphi List and 4 Additional Items).

		(i) Research Design							(ii) Participants Recruitment		(iii) Intervention Protocol and Follow-Up		(iv) Statistical Appropriateness and Outcome Reporting		Total
		Item 1	Item 2	Item 3	Item 5	Item 6	Item 7	Item 9	Item 4	Item 10	Item 11	Item 12	Item 8	Item 13	
No.	Study	Randomization Reported	Allocation Concealment	Baseline Comparability	Participants Blinding	Providers Blinding	Assessors Blinding	ITT Analysis	Eligibility Criteria	Sample Size Justification	Follow-Up Reported	Trial Registration	Variability Reported	Statistical Appropriateness	
1	Bieleninik, 2017 [47]	1	1	1	0	0	1	1	1	0	1	1	1	1	10
2	Corbett, 2019 [48]	1	1	1	0	0	0	0	1	0	0	1	1	1	7
3	Ding, 2022 [49]	1	0	0	0	0	0	0	0	0	0	0	0	0	1
4	Fu, 2024 [50]	1	0	1	1	0	1	0	1	1	0	0	1	1	8
5	Imankhah, 2018 [51]	1	0	0	0	0	0	0	1	0	0	0	1	0	3
6	Ioannou, 2020 [52]	1	1	1	0	0	0	0	1	0	0	1	1	1	7
7	Liu, 2024 [53]	1	0	1	0	0	0	0	1	0	0	0	0	0	3
8	Mössler, 2020 [54]	1	1	1	0	0	1	0	1	0	1	1	1	1	9
9	Rabeyron, 2020 [55]	1	0	0	1	0	0	0	1	1	0	1	1	1	7
10	Rahimi, 2021 [56]	1	0	0	0	0	0	0	1	0	1	0	1	1	5
11	Sabet, 2021 [57]	1	0	0	0	0	0	0	1	0	0	0	1	0	3
12	Sharda, 2018 [58]	1	1	1	0	0	1	0	1	0	0	1	1	1	8
